# Supplementary material for: Causal Relationship between Aging and Anorexia Nervosa: A White-Matter-Microstructure-Mediated Mendelian Randomization Analysis
Source: Biomedicines. 2024 Aug 16;12(8):1874. doi: 10.3390/biomedicines12081874 (PMC11351342; doi:10.3390/biomedicines12081874)
Supplement: Supplementary file 1 [file biomedicines-12-01874-s001.zip › Supplementary Figure/Figure S4/Singe SNP plot of MR analysis for TL to ubm-b-1614.pdf]

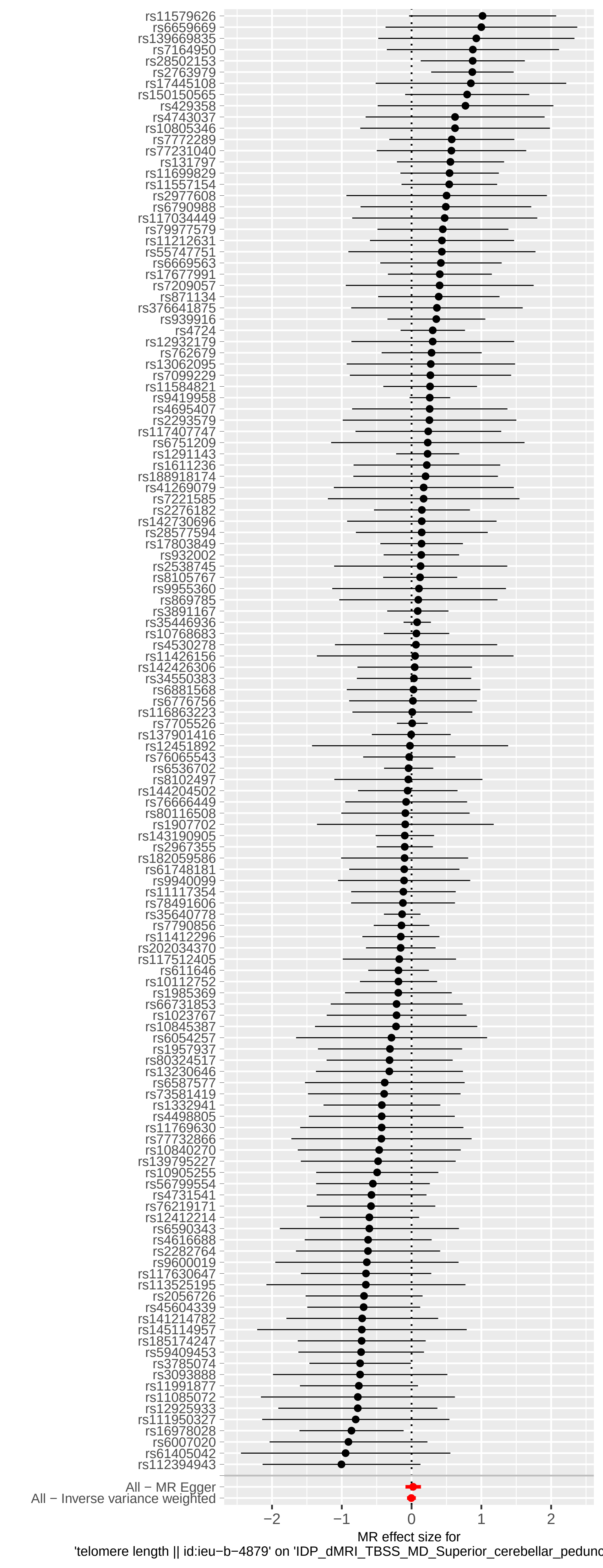

rs11579626
rs6659669
rs139669835
rs7164950
rs28502153
rs2763979
rs17445108
rs150150565
rs429358
rs4743037
rs10805346
rs7772289
rs77231040
rs131797
rs11699829
rs11557154
rs2977608
rs6790988
rs117034449
rs79977579
rs11212631
rs55747751
rs6669563
rs17677991
rs7209057
rs871134
rs376641875
rs939916
rs4724
rs12932179
rs762679
rs13062095
rs7099229
rs11584821
rs9419958
rs4695407
rs2293579
rs117407747
rs6751209
rs1291143
rs1611236
rs188918174
rs41269079
rs7221585
rs2276182
rs142730696
rs28577594
rs17803849
rs932002
rs2538745
rs8105767
rs9955360
rs869785
rs3891167
rs35446936
rs10768683
rs4530278
rs11426156
rs142426306
rs34550383
rs6881568
rs6776756
rs116863223
rs7705526
rs137901416
rs12451892
rs76065543
rs6536702
rs8102497
rs144204502
rs76666449
rs80116508
rs1907702
rs143190905
rs2967355
rs182059586
rs61748181
rs9940099
rs11117354
rs78491606
rs35640778
rs7790856
rs11412296
rs202034370
rs117512405
rs611646
rs10112752
rs1985369
rs66731853
rs1023767
rs10845387
rs6054257
rs1957937
rs80324517
rs13230646
rs6587577
rs73581419
rs1332941
rs4498805
rs11769630
rs77732866
rs10840270
rs139795227
rs10905255
rs56799554
rs4731541
rs76219171
rs12412214
rs6590343
rs4616688
rs2282764
rs9600019
rs117630647
rs113525195
rs2056726
rs45604339
rs141214782
rs145114957
rs185174247
rs59409453
rs3785074
rs3093888
rs11991877
rs11085072
rs12925933
rs111950327
rs16978028
rs6007020
rs61405042
rs112394943
All − MR Egger
All − Inverse variance weighted
−2
−1
0
1
2
MR effect size for
'telomere length || id:ieu−b−4879' on 'IDP_dMRI_TBSS_MD_Superior_cerebellar_peduncl
